# Evaluation of the rheumatoid arthritis susceptibility loci *HLA-DRB1*, *PTPN22*, *OLIG3/TNFAIP3*, *STAT4 *and *TRAF1/C5 *in an inception cohort

**DOI:** 10.1186/ar2969

**Published:** 2010-03-30

**Authors:** Ann W Morgan, James I Robinson, Philip G Conaghan, Stephen G Martin, Elizabeth MA Hensor, Michael D Morgan, Lori Steiner, Henry A Erlich, Hock-Chye Gooi, Anne Barton, Jane Worthington, Paul Emery

**Affiliations:** 1 NIHR - Leeds Musculoskeletal Biomedical Research Unit, Leeds Institute of Molecular Medicine, University of Leeds, Chapel Allerton Hospital, Chapeltown Road, Leeds LS7 4SA, UK; 2 Roche Molecular Systems, 4300 Hacienda Drive, Pleasanton, CA 94588-8566, USA; 3 Clinical Immunology, St James's University Hospital, Beckett Street, Leeds LS97TF, UK; 4 arc-Epidemiology Unit, Stopford Building, The University of Manchester, Oxford Road, Manchester M13 9PT, UK

## Abstract

**Introduction:**

This study investigated five confirmed rheumatoid arthritis (RA) susceptibility genes/loci (*HLA-DRB1*, *PTPN22*, *STAT4*, *OLIG3/TNFAIP3 *and *TRAF1/C5*) for association with susceptibility and severity in an inception cohort.

**Methods:**

The magnitude of association for each genotype was assessed in 1,046 RA subjects from the Yorkshire Early RA cohort and in 5,968 healthy UK controls. Additional exploratory subanalyses were undertaken in subgroups defined by autoantibody status (rheumatoid factor and anti-cyclic citrullinated peptide) or disease severity (baseline articular erosions, Health Assessment Questionnaire (HAQ) score and swollen joint count (SJC)).

**Results:**

In the total RA inception cohort, the *HLA-DRB1 *shared epitope (per-allele odds ratio (OR) = 2.1, trend *P *< 0.0001), *PTPN22 *(per-allele OR = 1.5, trend *P *< 0.0001), *OLIG3/TNFAIP3 *locus (per-allele OR = 1.2, trend *P *= 0.009) and *TRAF1/C5 *locus (per-allele OR = 1.1, trend *P *= 0.04) were associated with RA. The magnitude of association for these loci was increased in those patients who were autoantibody-positive. *PTPN22 *was associated with autoantibody-negative RA (per-allele OR = 1.3, trend *P *= 0.04). There was no evidence of association between these five genetic loci and baseline erosions or SJC in the total RA cohort, after adjustment for symptom duration. *TRAF1/C5 *was significantly associated with baseline HAQ, however, following adjustment for symptom duration (*P *trend = 0.03).

**Conclusions:**

These findings support the mounting evidence that different genetic loci are associated with autoantibody-positive and autoantibody-negative RA, possibly suggesting that many of the genes identified to date are associated with autoantibody production. Additional studies with a specific focus on autoantibody-negative RA will be needed to identify the genes predisposing to this RA subgroup. The *TRAF1/C5 *locus in particular warrants further investigation in RA as a potential disease severity locus.

## Introduction

Rheumatoid arthritis (RA) [MIM 180300] is a phenotypically heterogeneous, chronic destructive inflammatory disease of synovial joints, with an estimated prevalence of 0.8% in the UK [1]. A strong genetic component has been determined with heritability estimates of 50 to 60% from twin studies, with up to an additional 50% contribution from environmental and/or physiological risk factors [2]. Approximately 40% of genetic susceptibility to RA is accounted for by the *HLA-DRB1 *alleles encoding the shared epitope (SE), the major RA susceptibility locus [3], together with the protein tyrosine phosphatase nonreceptor 22 gene (*PTPN22*), a second susceptibility gene confirmed in populations of northern European ancestry [4]. Recent genome-wide association studies and candidate gene studies in RA have been highly successful in both the confirmation of known genetic associations and in highlighting new loci/immunological pathways that warrant further investigation [5]. The present study focuses on five confirmed RA susceptibility genes/loci - *HLA-DRB1 *(6p21), *PTPN22 *(1p13), *OLIG3/TNFAIP3 *(6q23), *STAT4 *(2q32) and *TRAF1/C5 *(9q33) - that are associated with RA with low-to-moderate risk in UK patients [6-8]. Some of these loci have been replicated in other Caucasian populations of northern European descent (reviewed in [5,9]), although only *HLA-DRB1 *SE and *STAT4 *have been confirmed in Asian populations [10-12].

RA is characterised by the presence of autoantibodies (rheumatoid factor (RF) and cyclic citrullinated peptide (CCP) antibodies) in a significant majority of patients. Many of the RA susceptibility genes identified to date appear to only be significant in the autoantibody-positive cohorts, although this may be secondary to the increased statistical power in this more prevalent patient subgroup [13]. If confirmed, this observation may suggest that these loci are influencing susceptibility to autoantibody production, perhaps through the loss of self-tolerance, thus explaining their association with multiple autoimmune disorders. The challenge over the next few years will be to identify whether these genes also influence the inflammatory process in RA *per se*. Unravelling the stage in the disease process in which these genes exert their maximum influence on RA pathogenesis will be necessary to fully unveil their clinical significance and reveal those pathways that are potential therapeutic targets or may become clinically useful biomarkers.

In the present study we sought to identify which parts of the RA pathway were affected by these susceptibility genes by studying an RA inception cohort from the UK. The Yorkshire Early Arthritis Register (YEAR) Consortium has made a considerable effort to review all patients presenting with early inflammatory symptoms within the community through the establishment of a rapid access system. This enables the full spectrum of RA to be studied. Confirmation of association in this cohort of newly diagnosed RA would support a contribution of these loci to RA susceptibility *per se *rather than disease persistence and severity, which potentially confound assessment of genetic susceptibility in cross-sectional secondary care cohorts. Further analyses, albeit at reduced statistical power, would determine whether the association is observed within both the autoantibody-positive (RF or CCP) and the autoantibody-negative (RF or CCP) subgroups, to determine whether the primary association was with autoantibody-positive disease. These two antibodies are highly correlated and much larger cohorts would be required to tease out antibody-specific effects.

We then sought to examine the influence of these loci on disease severity. Analysis of RA severity following the initiation of therapy is fraught with many difficulties since the physician's choice of medication, dose prescribed, toxicities, co-morbidities and psychosocial factors may all influence treatment outcome and persistence of inflammation. We therefore took advantage of our rapid access referral system to assess the influence of these loci on objective markers of joint inflammation (swollen joint count (SJC)), function (Health Assessment Questionnaire (HAQ)) and a measure of articular damage (erosions on plain radiographs of the hands and feet) at RA diagnosis. Although these secondary severity analyses would be of lower statistical power and require replication, we propose that any positive results would reveal those pathophysiological pathways that warrant further investigation to see whether they can be exploited as potential therapeutic targets and prognostic and/or predictive biomarkers that may ultimately guide therapeutic decisions.

## Materials and methods

### Sample collections

The YEAR network made a considerable effort to review all patients presenting with early inflammatory joint symptoms to ensure that the full spectrum of established RA could be studied. All cases fulfilled the 1987 American College of Rheumatology classification criteria for RA (n = 1,046), were Caucasian of Northern European descent, 18 years of age or older at disease onset and had a disease duration of less than 3 months (inception cohort). Comprehensive and standardised clinical documentation was undertaken for all patients at baseline. Radiographs of the hands and feet were obtained at presentation. The presence or absence of articular erosions, using standard clinical criteria, was documented by a radiologist at the treating hospital. Healthy controls (n = 5,968) were recruited from five centres in the UK, as described previously [8]. All participants were recruited after providing informed consent and the study was approved by the Multi-Centre Research Ethics Committee.

### Immunoassays

RA cases were recruited from NHS Rheumatology Clinics and the IgM RF status was measured using standard nephelometric assays. Patients who had ever had a titre ≥ 40 units/ml were defined as RF-positive. The presence of IgG CCP antibodies was documented at a single time point for a proportion of the patients (n = 619), using the commercially available DIASTAT™ anti-CCP ELISA (Axis-Shield Diagnostics Limited, Kimbolton, UK) or the ELiA CCP kit on an ImmunoCAP 100 (Phadia AB, Uppsala, Sweden). Patients who had a titre ≥ 5.5 units/ml or ≥ 10 units/ml for the two assays, respectively, were defined as CCP-positive.

### Genotyping

HLA-DRB1*01-16 types were determined at each centre using commercially available semi-automated PCR sequence-specific oligonucleotide probe typing techniques, as previously described [8]. In a subgroup of RA subjects, *HLA-DRB1 *typing was undertaken using a research assay based on PCR sequence-specific oligonucleotide probe linear array technology, developed by Roche Molecular Systems, Inc. (Pleasanton, CA, USA). PCR products amplified with biotinylated primers were denatured and hybridised to an immobilised probe array. Labelled PCR products hybridised to specific probes were detected using streptavidin-horseradish peroxidase and a chromogenic substrate. The probe binding pattern for each sample was scanned and the *HLA-DRB1 *genotype was assigned by the inhouse software StripScan. The *HLA-DRB1 *SE susceptibility sequences were defined as those encoding the amino acids LQKAA, LQRAA and LRRRA at positions 67, 70, 71, 73 and 74 in the third hypervariability region, and included HLA-DRB1*0101, HLA-DRB1*0102, HLA-DRB1*0401, HLA-DRB1*0404, HLA-DRB1*0405, HLA-DRB1*0408 and HLA-DRB1*1001.

Genotyping of the *PTPN22*, *OLIG3/TNFAIP3*, *STAT4 *and *TRAF1/C5 *loci was undertaken using the Sequenom MassArray platform, as described previously [6-8]. Genotyping was undertaken sequentially and these samples comprised a subgroup of the RA cases recruited by the UK Rheumatoid Arthritis Genetics (UKRAG) Consortium. For each individual plex, only those DNA samples with >90% success rate for all SNPs and those SNPs with >95% success rates across all samples passed the quality control step and were included in subsequent analyses. For some samples, inadequate DNA was available to enable inclusion in all plexes for this study. Some additional *PTPN22 *typing was undertaken using multi-locus SNP genotyping, whereby PCR products were hybridised to a linear probe array panel, which was developed by Roche Molecular Systems, Inc. Duplicate genotyping data were available for *PTPN22 *for >600 samples that corresponded perfectly, thus validating the latter assay. For each of the four non-MHC loci, the most significantly associated SNP identified to date in the UK population was tested (*PTPN22 *[rs2476601], *OLIG3/TNFAIP3 *locus [rs6920220], *STAT4 *[rs7574865] and *TRAF1/C5 *locus [rs10760130]).

### Analysis

Statistical analyses were performed using the SPSS 13.0 statistical package for Windows, (Chicago, IL, USA). Genotype frequencies were initially compared using 3 × 2 tables followed by a trend test, which performs well under either additive, dominant or recessive models. This is asymptotically equivalent to logistic or linear regression based on allele count (0,1,2). Two-sided *P *< 0.05 values are highlighted, although positive findings should be considered in light of the number of statistical tests undertaken and further studies in additional inception cohorts will be required to confirm these findings. The data were further stratified according to the presence of autoantibodies (RF and/or CCP). Subjects were grouped into autoantibody (RF or CCP)-positive and autoantibody-negative (RF and CCP) cohorts. Those individuals only positive for only a single antibody (RF^+^CCP^- ^and RF^-^CCP^+^) were thus coded as autoantibody-positive. Where data were only available for a single antibody (n = 445), those subjects were included in the autoantibody-positive or autoantibody-negative cohorts based on the data available. In the analysis of disease susceptibility, odds ratios (OR) and their 95% confidence intervals were calculated; the per-allele ORs are shown unless otherwise stated. The severity analyses were undertaken in a subgroup of patients where the symptom duration was known and <24 months. Logistic (presence/absence of erosions) and linear regression (SJC, HAQ) were used to undertake trend tests of association, which were adjusted for symptom duration.

## Results

The aim of the current study was to determine whether the previously reported RA susceptibility genes/loci (*HLA-DRB1*, *PTPN22*, *OLIG3/TNFAIP3*, *STAT4 *and *TRAF1/C5*) were associated with RA in an inception cohort. Additional exploratory subanalyses were undertaken to explore whether any positive findings were associated with a specific subgroup defined by autoantibody status or disease severity (baseline articular erosions, SJC and HAQ).

### Characteristics of the RA patients and controls

The RA cohort comprised 1,046 early RA patients, of whom 67% were female, 65% were RF-positive (n = 970), 63% were anti-CCP-positive (n = 619) and 70% of 968 subjects were *HLA-DRB1 *SE-positive. The disease characteristics of the total RA cohort and the autoantibody-positive (RF or CCP) and autoantibody-negative (RF and CCP) cohorts are presented in Table 1.

**Table 1:** Clinical characteristics of rheumatoid arthritis cases

	Total RA cohort (n = 1,046)		Autoantibody-positive RA (n = 692)		Autoantibody-negative RA (n = 325)	
			
	Number of valid subjects	Summary	Number of valid subjects	Summary	Number of valid subjects	Summary
Age (years)	926	59 (14)	411	57 (13)	183	60 (14)
Gender (female)	1046	702 (67%)	692	468 (68%)	325	210 (65%)
Rheumatoid factor-positive	970	628 (65%)	667	628 (94%)	N/A	N/A
Cyclic citrullinated peptide-positive	619	389 (63%)	445	389 (87%)	N/A	N/A
Erosions at baseline^a^	607	132 (22%)	421	95 (23%)	186	37 (28%)
Swollen joint count at baseline^a^	648	9.7 (6.2 to 10.2)	447	9.3 (8.8 to 9.9)	186	10.8 (9.8 to 11.8)
Health Assessment Questionnaire at baseline^a^	648	1.33 (1.27 to 1.39)	447	1.35 (1.28 to 1.42)	186	1.30 (1.19 to 1.41)

Data presented as mean (standard deviation), *n *(%) or mean (95% confidence interval). N/A, not applicable; RA, rheumatoid arthritis. ^a^Subgroup of individuals with documented symptom duration <24 months.

### Association of *HLA-DRB1 SE*, *PTPN22*, *OLIG3/TNFAIP3*, *STAT4 *and *TRAF1/C5 *with RA susceptibility

In the total RA inception cohort (n = 1,046), an initial model-free analysis of genotype frequencies was performed by undertaking a 3× 2 analysis. The global *P *values for each genetic locus are as follows: *HLA-DRB1 *SE alleles, *P *< 1 × 10^-6 ^(n = 968); *PTPN22*, *P *= 2 × 10^-6 ^(n = 855); *OLIG3/TNFAIP3*, *P *= 0.030 (n = 810); *STAT4*, *P *= 0.035 (n = 767); and *TRAF1/C5*, *P *= 0.041 (n = 763).

In subsequent analyses, trend tests were performed across all three genotypes; the ORs (95% confidence intervals) shown are per copy of the *HLA-DRB1 *SE or per copy of the minor allele for the remaining loci (Table 2). Thus, *HLA-DRB1 *SE alleles (OR = 2.1 (1.9 to 2.4), *P *< 0.0001), *PTPN22 *(OR = 1.5 (1.3 to 1.8), *P *< 0.0001), *OLIG3/TNFAIP3 *(OR = 1.2 (1.0 to 1.4), *P *= 0.009) and *TRAF1/C5 *(OR = 1.1 (1.0 to 1.3), *P *= 0.035) were associated with RA. The magnitude of association for these loci was increased in those patients who were autoantibody-positive (*HLA-DRB1 *SE, OR = 2.9 (2.5 to 3.3), *P *< 0.0001; *PTPN22*, OR = 1.6 (1.3 to 1.9), *P *< 0.0001; *OLIG3/TNFAIP3*, OR = 1.3 (1.1 to 1.5), *P *< 0.0001; and *TRAF1/C5*, OR = 1.2 (1.0 to 1.3), *P *= 0.043), with weak evidence of association for *STAT4 *(OR = 1.2 (1.0 to 1.4), *P *= 0.048) in this subgroup. The size of the autoantibody-negative cohort was limited (n = 298), resulting in lower statistical power - although a significant association was observed with *PTPN22 *(OR = 1.3 (1.0 to 1.7), *P *= 0.039). There was no evidence, however, of association for the *HLA-DRB1 *SE (OR = 1.1 (0.9 to 1.4), *P *= 0.279), or for the *OLIG3/TNFAIP3 *(OR = 0.9 (0.7 to 1.2), *P *= 0.578), *STAT4 *(OR = 1.1 (0.9 to 1.4), *P *= 0.266) or *TRAF1/C5 *(OR = 1.1 (0.9 to 1.3), *P *= 0.392) loci in autoantibody-negative disease. A statistically significant difference in the genotype frequencies between the autoantibody-positive and autoantibody-negative cohorts was observed for *HLA-DRB1 *SE (trend *P *< 0.001) and *OLIG3/TNFAIP3 *(trend *P *= 0.008), but not for *PTPN22 *(trend *P *= 0.2), *STAT4 *(trend *P *= 0.8) or *TRAF1/C5 *(trend *P *= 0.6).

**Table 2:** Genotype frequencies in rheumatoid arthritis, and in autoantibody-positive/autoantibody-negative rheumatoid arthritis, relative to controls

RA cohort	Controls, *n *(%)	Cases, *n *(%)	Per-allele OR (95% CI)	*P *trend	Autoantibody-positive RA	Per-allele OR (95% CI)	*P *trend	Autoantibody-negative RA	Per-allele OR (95% CI)	*P *trend
*HLA-DRB1 *shared epitope	n = 1,351	n = 968			n = 643			n = 298		
Negative	717 (53.1)	290 (30.0)			138 (21.5)			144 (48.3)		
One copy	517 (38.3)	475 (49.1)	2.12 (1.87 to 2.40)	<0.0001	332 (51.6)	2.85 (2.46 to 3.29)	<0.0001	129 (43.3)	1.11 (0.92 to 1.35)	0.279
Two copies	117 (8.7)	203 (21.0)			173 (26.9)			25 (8.4)		
*PTPN22 *(rs2476601)	n = 3,492	n = 855			n = 572			n = 259		
GG	2,817 (80.7)	624 (73.0)			413 (72.2)			195 (75.3)		
GA	635 (18.2)	212 (24.8)	1.50 (1.28 to 1.75)	<0.0001	144 (25.2)	1.56 (1.31 to 1.87)	<0.0001	60 (23.2)	1.32 (1.01 to 1.72)	0.039
AA	40 (1.1)	19 (2.2)			15 (2.6)			4 (1.5)		
*OLIG3/TNFAIP3 *(rs6920220)	n = 3,478	n = 810			n = 545			n = 242		
GG	2,146 (61.7)	464 (57.3)			294 (53.9)			155 (64.0)		
GA	1,197 (34.4)	303 (37.4)	1.19 (1.04 to 1.35)	0.009	220 (40.4)	1.32 (1.14 to 1.54)	<0.0001	77 (31.8)	0.94 (0.74 to 1.18)	0.578
AA	135 (3.9)	43 (5.3)			31 (5.7)			10 (4.1)		
*STAT4 *(rs7574865)	n = 3,520	n = 767			n = 515			n = 234		
GG	2,151 (61.1)	451 (58.8)			294 (57.1)			143 (61.1)		
GT	1,208 (34.3)	264 (34.4)	1.14 (1.00 to 1.29)	0.054	190 (36.9)	1.17 (1.00 to 1.36)	0.048	70 (29.9)	1.13 (0.91 to 1.41)	0.266
TT	161 (4.6)	52 (6.8)			31 (6.0)			21 (9.0)		
*TRAF1/C5 *(rs10760130)	n = 3,506	n = 763			n = 512			n = 233		
AA	1,096 (31.3)	224 (29.4)			151 (29.5)			69 (29.6)		
GA	1,742 (49.7)	363 (47.6)	1.13 (1.01 to 1.26)	0.035	238 (46.5)	1.15 (1.00 to 1.31)	0.043	114 (48.9)	1.09 (0.90 to 1.31)	0.392
GG	668 (19.1)	176 (23.1)			123 (24.0)			50 (21.5)		

CI, confidence interval; OR, odds ratio; RA, rheumatoid arthritis.

### Association of *HLA-DRB1 SE*, *PTPN22*, *OLIG3/TNFAIP3*, *STAT4 *and *TRAF1/C5 *with RA severity

In view of the potential importance of symptom duration on disease severity measures at baseline, the severity analyses were undertaken in a subgroup of 666 individuals for whom the symptom duration had been recorded and was <24 months, to allow adjustment for symptom duration in the regression models.

Prevalent erosions were observed in 21.5% of 615 subjects at presentation and were associated with symptom duration (*P *= 0.011), but not with SJC (*P *= 0.96, n = 595), RF (*P *= 0.21, n = 580) or CCP (*P *= 0.19, n = 428). Likewise, SJC was associated with symptom duration (*P *< 0.001, n = 666), but not RF (*P *= 0.07, n = 623) or CCP (*P *= 0.15, n = 459). Finally, HAQ was associated with symptom duration (*P *= 0.001, n = 666) but not with CCP (*P *= 0.66, n = 480), with only a weak trend toward significance for RF (*P *= 0.05, n = 633). There was no evidence that *HLA-DRB1 *SE, *PTPN22*, *OLIG3/TNFAIP3*, *STAT4 *or *TRAF1/C5 *were significantly associated with prevalent erosions in logistic regression analyses of the total RA cohort, after adjustment for symptom duration (Table 3).

**Table 3:** Association with disease severity measures in early rheumatoid arthritis and in autoantibody-positive/antibody-negative disease

RA cohort	Erosions			Swollen joint count			Health Assessment Questionnaire		
			
	Erosive, *n *(%)	Per-allele OR (95% CI)	***P *trend adj.**^**a**^	Mean (95% CI)	Coefficient (95% CI)	***P *trend adj.**^a^	Mean (95% CI)	Coefficient (95% CI)	***P *trend adj.**^a^
*HLA-DRB1 *shared epitope	554 (21.5)			n = 587			n = 587		
Negative	175 (22.9)	0.88	0.398	10.5 (9.5 to 11.5)	-0.03	0.455	1.35 (1.24 to 1.47)	-0.00	0.965
One copy	278 (20.9)	(0.65 to 1.19)		9.4 (8.7 to 10.1)	(-1.04 to 0.47)		1.38 (1.29 to 1.46)	(-0.09 to 0.09)	
Two copies	101 (20.8)			9.7 (8.4 to 11.0)			1.27 (1.14 to 1.41)		
*PTPN22 *(rs2476601)	580 (20.7)			n = 468			n = 468		
GG	423 (22.0)	0.75	0.189	9.5 (8.8 to 10.2)	-0.02	0.593	1.32 (1.23 to 1.40)	0.06	0.133
GA	147 (17.0)	(0.48 to 1.16)		9.0 (7.8 to 10.1)	(-1.31 to 0.75)		1.43 (1.30 to 1.56)	(-0.03 to 0.21)	
AA	10 (20.0)			10.4 (1.9 to 18.9)			1.48 (0.35 to 2.61)		
*OLIG3/TNFAIP3 *(rs6920220)	551 (20.3)			n = 468			n = 468		
GG	302 (19.9)	1.03	0.853	9.4 (8.6 to 10.1)	0.04	0.291	1.36 (1.27 to 1.45)	-0.01	0.880
GA	217 (20.7)	(0.73 to 1.46)		9.3 (8.3 to 10.2)	(-0.39 to 1.31)		1.36 (1.24 to 1.48)	(-0.11 to 0.09)	
AA	32 (21.9)			10.5 (7.7 to 13.3)			1.07 (0.76 to 1.37)		
*STAT4 *(rs7574865)	476 (20.4)			n = 468			n = 468		
GG	271 (22.1)	0.82	0.291	9.5 (8.7 to 10.2)	0.01	0.873	1.38 (1.28 to 1.47)	-0.06	0.193
GT/	170 (18.2)	(0.57 to 1.19)		9.3 (8.2 to 10.3)	(-0.82 to 0.97)		1.35 (1.23 to 1.46)	(-0.17 to 0.04)	
TT	35 (17.1)			9.5 (7.0 to 12.0)			1.12 (0.85 to 1.39)		
*TRAF1/C5 *(rs10760130)	474 (20.3)			n = 468			n = 468		
AA	140 (16.4)	1.18 (0.86 to 1.61)	0.311	9.7 (8.6 to 10.8)	-0.03	0.502	1.25 (1.11 to 1.38)	0.09	0.031
GA	225 (22.7)			9.1 (8.3 to 9.9)	(-1.07 to 0.52)		1.34 (1.24 to 1.43)	(0.01 to 0.19)	
GG	109 (20.2)			9.6 (8.4 to 10.8)			1.50 (1.35 to 1.65)		

CI, confidence interval; OR, odds ratio; RA, rheumatoid arthritis. ^a^Adjusted for symptom duration (all cases having a symptom duration <24 months).

There was also no evidence of association between these five genetic loci with SJC, after adjustment for symptom duration in linear regression analyses of the total RA cohort (Table 3). The *TRAF1/C5 *locus, however, was significantly associated with HAQ, following adjustment for symptom duration (*P *= 0.031, n = 468). It is important to note that raw *P *values have been presented throughout. These findings should therefore be considered in light of the number of statistical tests undertaken in the analysis of RA severity, and further larger studies will be required to substantiate these findings. If a strict Bonferroni correction was applied for the severity analyses, the level of significance would be set at *P *< 0.003; and for the combined susceptibility and severity studies, the level would be set at *P *< 0.002.

## Discussion

In the present study we confirmed that the *HLA-DRB1 *SE, *PTPN22 *and the *OLIG3/TNFAIP3 *and *TRAF1/C5 *loci were associated with susceptibility to RA in an inception cohort. The effect sizes were comparable with the total UKRAG cohort (*HLA-DRB1 *SE (2.1, 2.6), *PTPN22 *(1.5, 1.5), *OLIG3/TNFAIP3 *(1.2, 1.2) and *TRAF1/C5 *loci (1.1, 1.1); early RA compared with total UKRAG cohort [6-8,14], respectively). The distinction between susceptibility and severity remains difficult and some may argue that the present study still does not sample the full spectrum of RA observed in the community. We believe, however, these findings do support a genuine association with RA susceptibility. Further studies utilising community-based cohorts will ultimately be required to confirm these findings. Of particular interest is the observation that for each of these loci the association was most marked in the autoantibody-positive subgroup and, although the association with *STAT4 *was not confirmed, some evidence of a weak association was observed in the subgroup harbouring autoantibodies. Indeed, only *PTPN22 *demonstrated any suggestion of association with RA in the autoantibody-negative cohort, which warrants further investigation in a larger inception cohort.

These findings support the mounting evidence that different genetic loci are associated with autoantibody-positive and autoantibody-negative RA. Many of the genes identified to date may therefore predispose to autoimmunity in general, or more specifically the immunological processes involved in the breakdown of self-tolerance and autoantibody production. This is supported by the association of *PTPN22 *and the *OLIG3/TNFAIP3 *locus with other autoantibody-associated autoimmune diseases, such as systemic lupus erythematosus, Graves disease and type 1 diabetes [5,7,8,15-17], but not with those autoimmune/inflammatory disorders not associated with autoantibody production, such as ulcerative colitis, Crohn's disease and ankylosing spondylitis [5,18,19].

The transcription factor encoded by *STAT4 *is downstream of several cytokines that play a crucial role in the development of Th1 and Th17 responses, such as IL-12, IL-15 and IL-23 [20,21] and the type I interferons [21,22]. Whilst this gene undoubtedly contributes to susceptibility to some autoantibody-associated diseases (RA, systemic lupus erythematosus and type I diabetes), there are recent reports that it may be associated with both clinical forms of inflammatory bowel diseases [23] - suggesting that rather than contributing to autoantibody production, it may be a common risk factor for inflammatory disease *per se*. This is consistent with the apparent association of *STAT4 *with both autoantibody-positive and autoantibody-negative RA in the literature [7,11,15,23], although the numbers were considerably lower for the latter subgroup, in all reported series.

Although we acknowledge there was also reduced power to detect trends in the autoantibody-negative cohort in the current study, we were unable to see any suggestive evidence of association with *STAT4*, with no substantive skewing of allele or genotype frequencies. Additional studies with increased power to investigate autoantibody-negative RA will be needed to unravel the genes predisposing to RA in this patient subgroup. Power calculations based on replicating the association of *PTPN22 *with autoantibody-negative disease with an effect size in the range 1.2 to 1.5 revealed that 2,909 cases would be required if one control per case was used, reducing to 2,169 if two controls per case were used. If all five loci were investigated, the number of autoantibody-negative cases required would need to increase to 4,329 and 3,227, respectively - with ~12,000 cases required if the effect size was reduced to 1.1. Such studies will require a concerted international effort and large-scale recruitment of cases from around the globe.

In the current study, we analysed the SNPs displaying the strongest association with RA, after the strongly associated *HLA-DRB1 *SE alleles, in the UK Caucasian population [24]. To date, there have been relatively few genetic loci that have shown consistent association with disease severity in RA. As stated previously, it is very difficult to tease out the influence of drugs and other nongenetic factors when designing these studies. We therefore chose to investigate these susceptibility loci with objective markers of joint inflammation or disease severity (radiographic erosions, SJC, and HAQ) at presentation to the rheumatology department. The presence of radiographic articular erosions is generally accepted as the most objective measure of articular damage that is accessible for all rheumatologists, but there is currently a paucity of studies investigating this early time point.

In this exploratory study we were unable to find any strong evidence that these five susceptibility genes were associated with disease severity measures at baseline. We found some weak evidence to support the association of the *TRAF1/C5 *locus with HAQ at baseline, a marker of function, although this would not remain significant after correction for multiple comparisons. Carriers of the minor A allele of rs10818488 at this locus were previously shown to have increased radiographic progression, albeit in a small cohort of 278 CCP-positive individuals [25], whereas homozygosity for the G allele may be associated with mortality [26]. We found no evidence, however, that *TRAF1/C5 *was associated with erosions in this cohort. It is important to note that due to a high level of linkage disequilibrium between the genes encoding TNF receptor-associated factor 1 and complement component 5, it is currently not possible to unravel which of these two genes at 9q33.2 harbours the causal variant. Both are excellent candidate genes that may perpetuate chronic inflammation in RA. TNF receptor-associated factor 1 is a negative regulator of TNF receptor and Toll-like receptor signalling, and may contribute to the proliferation of T cells, and the complement pathway may directly contribute to immune complex-mediated inflammation [25,27]. Intensive resequencing efforts with additional fine-mapping studies in expanded genetic cohorts are currently underway to identify the disease-associated variant at such loci. It is likely that for those loci with very strong and extensive linkage disequilibrium, however, functional studies may ultimately be required to unravel the biological significance of the genetic findings.

## Conclusions

The importance of studying inception cohorts cannot be underestimated since most hospital-based cohorts represent the severe end of the RA spectrum and genetic associations derived from such patients cannot discriminate between susceptibility and severity factors. The present study supports an association between *HLA-DRB1 *SE alleles, *PTPN22*, *OLIG3/TNFAIP3*, *STAT4 *and *TRAF1/C5 *with susceptibility to autoantibody-positive RA, but only the *TRAF1/C5 *locus was associated with disease severity in this cohort, although this would no longer remain significant after correction for multiple comparisons. Substantially larger cohorts will ultimately be required to have adequate power to investigate clinical, immunological and genetic factors determining disease severity and response to therapy, and we hope these data will assist future power calculations. Although the ultimate goal is the development of clinical algorithms that will facilitate personalised medicine, it also remains important to identify the immunological and biochemical pathways that determine outcome, which can then be targeted therapeutically. The *TRAF1/C5 *locus in particular warrants further investigation as a potential disease severity locus in RA.

## Abbreviations

CCP: cyclic citrullinated peptide; HAQ: Health Assessment Questionnaire; IL: interleukin; OR: odds ratio; PCR: polymerase chain reaction; RA: rheumatoid arthritis; RF: rheumatoid factor; SE: shared epitope; SJC: swollen joint count; SNP: single nucleotide polymorphisms; STAT: signal transducer and activator of transcription; Th: T-helper cell; TNF: tumour necrosis factor; UKRAG: UK Rheumatoid Arthritis Genetics; YEAR: Yorkshire Early Arthritis Register.

## Competing interests

LS and HAE are employed by Roche Molecular Systems, Inc. (Pleasanton, CA, USA), provider of *HLA-DRB1 *and *PTPN22 *genotyping reagents for a subgroup of subjects analysed in the present study. Otherwise the authors declare that they have no competing interests.

## Authors' contributions

AWM conceived and designed this study, contributed to the acquisition of data, undertook the statistical analyses, interpreted the data and wrote the manuscript. JIR contributed to the acquisition of data and drafted part of the manuscript. PGC contributed to the acquisition of data and critically reviewed the manuscript. SGM contributed to the acquisition of data and critically reviewed the manuscript. The YEAR Consortium contributed to the acquisition of data. EMAH contributed to the statistical analysis and critically reviewed the manuscript. MDM drafted part of the manuscript. LS developed the multi-locus SNP genotyping platform and critically reviewed the manuscript. HAE developed the multi-locus HLA-DRB1 genotyping platform and critically reviewed the manuscript. H-CG contributed to the acquisition of data and critically reviewed the manuscript. AB contributed to the acquisition of data and critically reviewed the manuscript. The UKRAG Consortium contributed to the acquisition of data and critically reviewed the manuscript. JW contributed to the acquisition of data and critically reviewed the manuscript. PE contributed to the acquisition of data and critically reviewed the manuscript.

## Authors' information

**Membership of the YEAR Consortium**.

**Management team: **Paul Emery, Philip Conaghan, Ann Morgan, Anne-Maree Keenan, Elizabeth Hensor and Julie Kitcheman at the Section of Musculoskeletal Disease, Leeds Institute of Molecular Medicine, University of Leeds, UK. Mark Quinn at York District Hospital, York, UK.

**Consultants: **Gareth Huston, Mike Martin, Colin Pease and Sally Cox at the Section of Musculoskeletal Disease, Leeds Institute of Molecular Medicine, University of Leeds, UK. Michael Green and Amanda Isdale at York District Hospital, York, UK. Andrew Gough and Michael Green at Harrogate District Hospital, Harrogate, UK. Richard Reece at the Huddersfield Royal Infirmary, Huddersfield, UK. Lesley Hordon at Dewsbury District and General Hospital, Dewsbury, UK. Philip Helliwell and Richard Melsom at St Luke's Hospital, Bradford, UK. Sheelagh Doherty at Hull Royal Infirmary, Hull, UK. Ade Adebajo at Barnsley District General Hospital, Barnsley, UK. Andrew Harvey, Steve Jarrett and Zunaid Karim at Pinderfields General Hospital, Wakefield, UK. Dennis McGonagle at Calderdale Royal Hospital, Halifax, UK.

**SpRs: **Victoria Bejarano and Jackie Nam at the Section of Musculoskeletal Disease, Leeds Institute of Molecular Medicine, University of Leeds, UK.

**Nurses: **Claire Brown, Christine Thomas, David Pickles, Alison Hammond, Belinda Rhys-Evans, Barbara Padwell, Sally Smith and Heather King at the Section of Musculoskeletal Disease, Leeds Institute of Molecular Medicine, University of Leeds, UK. Anne Gill and Julie Green at York District Hospital, York, UK. Beverley Neville at Harrogate District Hospital, Harrogate, UK. Alan Fairclough and Caroline Nunns at Huddersfield Royal Infirmary, Huddersfield, UK. Jill Firth and Linda Sigsworth at St Luke's Hospital, Bradford, UK. Jayne Heard at Hull Royal Infirmary, Hull, UK. Julie Madden and Lynda Taylor at Calderdale Royal Hospital, Halifax, UK.

**Laboratory staff: **Diane Corscadden, Karen Henshaw, Lubna-Haroon Rashid, Stephen G Martin and James I Robinson at the Section of Musculoskeletal Disease, Leeds Institute of Molecular Medicine, University of Leeds, UK.

**Membership of the UKRAG Consortium**.

**University of Manchester: **Stephen Eyre, Anne Hinks, Laura J Gibbons, John Bowes, Edward Flynn, Paul Martin, Wendy Thomson, Anne Barton and Jane Worthington at the arc-Epidemiology Unit, The University of Manchester, UK.

**University of Leeds: **The YEAR consortium, Stephen Martin, James I Robinson, Ann W Morgan and Paul Emery at the Leeds Institute of Molecular Medicine, Section of Musculoskeletal Disease, University of Leeds, UK.

**University of Sheffield: **Anthony G Wilson at the School of Medicine & Biomedical Sciences, The University of Sheffield, UK.

**University of London: **Sophia Steer at the Clinical and Academic Rheumatology, Kings College Hospital NHS Foundation Trust, London, UK.

**University of Aberdeen: **Lynne Hocking and David M Reid at the Bone Research Group, Department of Medicine & Therapeutics, University of Aberdeen, UK.

**University of Oxford: **Pille Harrison and Paul Wordsworth at University of Oxford Institute of Musculoskeletal Sciences, Oxford, UK.
